# The carbohydrate metabolism and expression of carbohydrate-active enzyme genes in *Aspergillus luchuensis* fermentation of tea leaves

**DOI:** 10.3389/fmicb.2024.1408645

**Published:** 2024-06-04

**Authors:** Ruoyu Li, Teng Wang, Nianguo Bo, Qi Wang, Qiuyue Chen, Zhengwei Liang, Yanhui Guan, Bin Jiang, Yan Ma, Ming Zhao

**Affiliations:** ^1^College of Tea Science, Yunnan Agricultural University, Kunming, Yunnan, China; ^2^College of Food Science and Technology, Yunnan Agricultural University, Kunming, Yunnan, China; ^3^State Key Laboratory for Conservation and Utilization of Bio-Resources in Yunnan, Yunnan Agricultural University, Kunming, Yunnan, China; ^4^The Key Laboratory of Medicinal Plant Biology of Yunnan Province, Yunnan Agricultural University, Kunming, Yunnan, China; ^5^National-Local Joint Engineering Research Center on Germplasm Innovation and Utilization of Chinese Medicinal Materials in Southwestern China, Yunnan Agricultural University, Kunming, Yunnan, China

**Keywords:** *Aspergillus luchuensis*, tea fermentation, carbohydrate, glycosides, CAZymes

## Abstract

**Introduction:**

Carbohydrates, which make up 20 to 25% of tea beverages, are responsible for their flavor and bioactivity. Carbohydrates of pu-erh tea change during microbial fermentation and require further research. In this study, we examined the carbohydrate metabolism and expression of carbohydrate-active enzyme genes during the fermentation of tea leaves with *Aspergillus luchuensis*.

**Methods:**

Widely targeted metabolomics analysis, high-performance anion-exchange chromatography measurements, and transcriptomics were used in this study.

**Results:**

After fermentation, the levels of soluble sugar, hemicellulose, lignin, eight monosaccharides, and seven sugar alcohols increased. Meanwhile, the relative contents of polysaccharides, D-sorbitol, D-glucose, and cellulose decreased. High expression of 40 genes encoding 16 carbohydrate enzymes was observed during fermentation (FPKM>10). These genes encode L-iditol 2-dehydrogenase, pectinesterase, polygalacturonase, α-amylase, glucoamylase, endoglucanase, β-glucosidase, β-galactosidase, α-galactosidase, α-glucosidase, and glucose-6-phosphate isomerase, among others.

**Discussion:**

These enzymes are known to break down polysaccharides and cell wall cellulose, increasing the content of monosaccharides and soluble sugars.

## 1 Introduction

Microorganisms play a crucial role in determining the metabolism and quality of fermented food (Wang et al., 2023). Fermented food has a fundamental microorganism that plays a vital role in enhancing its quality. For instance, lactic acid bacteria are commonly used in various dietary sources such as curd, pickles, milk, and wheat dough. They are also available in the form of supplements to improve flavor and overall quality (Khushboo et al., 2023). Similarly, *Aspergillus*, *Zygosaccharomyces*, *Candida*, and *Clavispora* contribute to the formation of rich soy sauce flavor (Feng et al., 2023). The production of enzymes by microorganisms significantly affects the metabolites in food. Recently, studies have reported the importance of *Lactobacillus gasseri* in producing fructooligosaccharides (de Lima et al., 2021) and *Monascus purpureus* in synthesizing esters (Xu et al., 2021). Microbial fermentation is the critical step in the manufacturing of pu-erh tea; *Aspergillus luchuensis* induces dramatic changes in the chemical composition and content of tea (Chen et al., 2022). It is worth studying the changes in metabolism caused by *A. luchuensis* during the fermentation process of ripened pu-erh tea (RPT).

RPT is a renowned traditional Chinese tea that is crafted exclusively in Yunnan Province, located in Southwest China. It is made by fermenting sun-dried green tea from fresh leaves of *Camellia sinensis* var. *assamica* using microbial fermentation (Huang et al., 2019). Recently, it has been reported that this tea has various health benefits, such as bacteriostatic properties, lipid-lowering effects (Jia et al., 2022), antioxidation, antimutation (Wang et al., 2022), and antitumor properties (Armstrong et al., 2020). The metabolism of carbohydrates in RPT during post-fermentation is attributed to the core microbiota and their activities. Bian et al.(2022) has discovered that monosaccharides like glucose, fructose, arabinose, ribose, and ribulose are consumed by microbes during early and middle fermentation stages, whereas carboxylic acids and other monosaccharides such as galactose, rhamnose, mannose, xylose, talose, allose, and galactinol accumulate during the middle and late pile-fermentation stages. In our previous study, we identified microbial carbohydrate-active enzymes (CAZymes) that are involved in the degradation of various polysaccharides such as cellulose, xylan, xyloglucan, pectin, starch, lignin, galactomannan, and chitin (Zhao et al., 2019). Recently, Ma et al. (2021) has detected CAZymes such as endoglucanases, glucosidases, and cellulases involved in the degradation of cellulose, starch, lignin, pectin, xylan, and xyloglucan during the fermentation of tea leaves by *Aspergillus niger*, *Aspergillus tamarii*, and *Aspergillus fumigatus*. During pu-erh tea fermentation, the impact of fungal species *A. luchuensis* on carbohydrate metabolism remains unclear, despite its significance in fermented food (Mageswari et al., 2016). Therefore, further investigation is required to establish a foundation for Pu-erh tea fermentation and the wider food fermentation industry.

High-performance liquid chromatography (HPLC), high-performance anion-exchange chromatography (HPAEC), and ultra-performance liquid chromatography–tandem mass spectrometry (UPLC–MS/MS) were used to study the carbohydrate metabolism of *A. luchuensis* in the pure culture fermentation of sterile sun-dried green tea. Expressions of fungal CAZymes genes were surveyed using transcriptomics. This study has advanced knowledge regarding CAZymes genes and carbohydrate metabolism in fungal fermentation of plant mass, especially on tea leaves.

## 2 Materials and methods

### 2.1 Pure culture fermentation of tea leaves and metabolomic analysis

Pure culture fermentation (FT) and control tests (CK) with inoculation with or without *A. luchuensis* in sterilized sun-dried green tea leaves were developed according to our previous study (Ma et al., 2022). Sterilized sun-dried green tea leaves were fermented for 16 d, and six tea samples were collected. Fermented tea leaves were subjected to Metware Biotechnology Co., Ltd. (Wuhan, China), and analyzed by ultra-high-performance liquid chromatography (UPLC) and tandem mass spectrometry (MS/MS) in a widely targeted metabolomics approach. The extraction of metabolites in tea leaves, UPLC separation, ESI-MS/MS monitoring, and data processing were performed as described previously (Ma et al., 2022).

### 2.2 Measurement of saccharides in tea leaves

Saccharides in fermented tea leaves were sent to Sanshu Biotechnology Co., Ltd. (www.sanshubio.com, Jiangsu, China) and analyzed by using a Thermo ICS-5000 (Dionex, Thermo Scientific, Waltham, US) ion chromatography system with an electrochemical detector; 5 mg of the sample was hydrolyzed with trifluoroacetic acid (2 M) at 121°C for 2 h in a sealed tube and dried with nitrogen. The sample was washed with methanol two to three times and then blow-dried. The residue was re-dissolved in deionized water and filtered through 0.22-μm microporous filtering film for measurement. The sample extracts were analyzed by HPAEC on a CarboPac PA-20 anion-exchange column (3 × 150 mm; Dionex) using a pulsed amperometric detector (PAD; Dionex ICS-5000 system). The flow rate was 0.5 mL/min, and the injection volume was 5 μL. The solvent system consisted of A (0.1 M NaOH) and B: (0.1 M NaOH, 0.2 M NaAc). The gradient program was 95:5 (v/v) at 0 min, 80:20 (v/v) at 30 min, 60:40 (v/v) at 30.1 min, 60:40 (v/v) at 45 min, 95:5 (v/v) at 45.1 min, and 95:5 (v/v) at 60 min.

### 2.3 Measurement of polysaccharides in tea leaves

Approximately 5 mg of the sample was hydrolyzed with trifluoroacetic acid (2 M) at 121°C for 2 h in a sealed tube. The extracts were dried with nitrogen and washed with methanol two to three times. The residue was re-dissolved in deionized water and filtered through a 0.22-μm microporous filtering film for measurement. The sample extracts were analyzed by HPAEC on a CarboPac PA-20 anion-exchange column (3 × 150 mm; Dionex) using a pulsed amperometry detector (PAD; Dionex ICS 5000 system). The flow rate was 0.5 mL/min, and the injection volume was 5 μL. The solvent system consisted of A (0.1 M NaOH) and B (0.1 M NaOH, 0.2 M NaAc). The gradient program was as follows: 95:5 (v/v) at 0 min, 80:20 (v/v) at 30 min, 60:40 (v/v) at 30.1 min, 60:40 (v/v) at 45 min, 95:5 (v/v) at 45.1 min, and 95:5 (v/v) at 60 min.

### 2.4 Expression of CAZymes genes in fermentation

The expression of CAZymes genes in fermentation was analyzed through a transcriptomics analysis. Fungal mycelia were collected and submitted to Gene Denovo Biotechnology Co., Ltd. (Guangzhou, China) to perform transcriptomics analysis of gene expression in fermentation. Detailed approaches are described in our previous report (Ma et al., 2022).

### 2.5 Data analysis

Principal component analysis (PCA) and orthogonal partial least-squares discriminant analysis (OPLS-DA) on the platform of MetaboAnalyst 5.0^1^ were used to compare the metabolite profiles and the sample compositions. Variable importance in projection (VIP) was used to rank the overall contribution of each variable in the OPLS-DA model, and variables with VIP > 1.0, *p* < 0.05 (*t*-test), and fold change (FC) of >2 or < 0.5 were considered differentially changed metabolites (DCMs). All experiments were repeated three times.

## 3 Results

### 3.1 Metabolism of polysaccharides

The content of soluble sugar increased from 2.35% ± 0.03 to 2.63% ± 0.02% after fermentation. The content of polysaccharides was 67.05 ± 2.77 mg/g in CK, and it decreased to 46.00 ± 1.70 mg/g after fermentation. Total lignin and hemicellulose were 14.95% ± 0.20 and 2.32% ± 0.06% in CK, which increased to 20.60% ± 0.45 and 2.87% ± 0.08%, respectively, in FT. However, the contents of cell wall cellulose decreased from 18.55% ± 0.16 to 16.72% ± 0.16% (Table 1). Sharma reported that some *Aspergillus* species have the capability to break down cellulose and other polysaccharides into lignin (Sharma Ghimire et al., 2016); this can explain the increase of lignin in fermentation.

**Table 1:** Content of polysaccharides in tea leaves.

Carbohydrates	CK	FT
Soluble sugar (%)	2.35 ± 0.03	2.63 ± 0.02*
Total lignin (%)	14.95 ± 0.20	20.6 ± 0.45*
Total hemicellulose (%)	2.32 ± 0.06	2.87 ± 0.08*
Cell wall cellulose (%)	18.55 ± 0.16	16.72 ± 0.16*
Polysaccharide (%)	6.71 ± 0.28	4.60 ± 0.17*

Content, mean ± SD (*n* = 2); significant difference compared with samples (*p < 0.05).

### 3.2 Metabolism of soluble sugars

**Figure 1:**
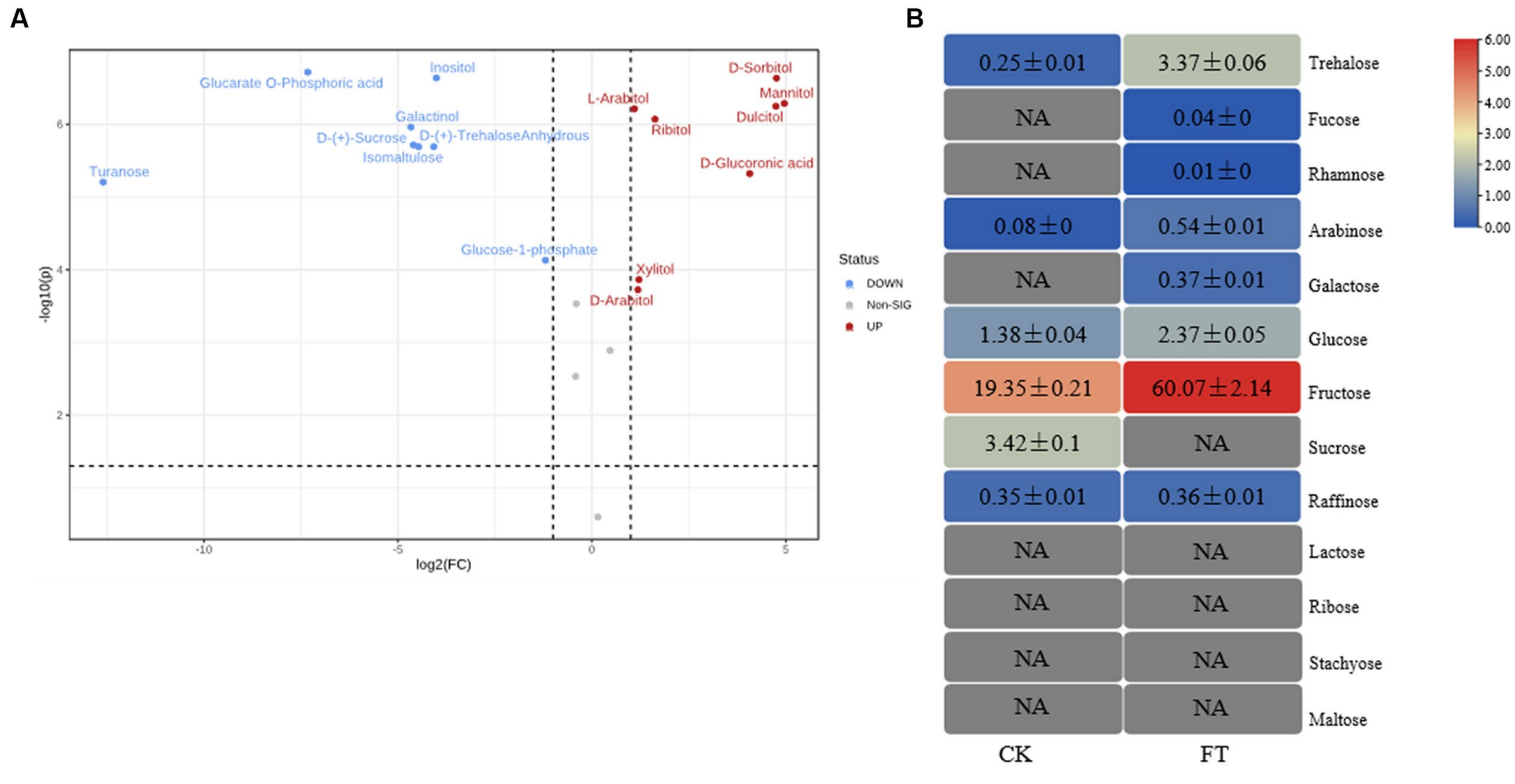
RLs of differentially changed monosaccharides by widely targeted metabolism **(A)** and the content of monosaccharides differentially changed by HPAEC **(B)** of CK and FT. “NA” indicates that the compound was not detected.

In total, 20 monosaccharide metabolites were identified by metabolomics (Supplementary Table S1). The relative levels (RLs) of 13 monosaccharides were significantly higher in FT (VIP > 1.0, *p* < 0.05, FC > 2) (Figure 1A), including mannitol, sorbitol, dulcitol, glucuronic acid, ribitol, xylitol, arabitol, arabitol, arabinose, trehalose-6-phosphate, glucose, gluconic acid, and glucose-1-phosphate. Among them, the RLs of eight monosaccharides increased more than 10-fold, including mannitol, sorbitol, dulcitol, glucuronic acid, ribitol, xylitol, arabitol, and arabinose. Meanwhile, the RLs of seven monosaccharides significantly decreased in FT (VIP > 1.0, *p* < 0.05, FC < 0.5), including inositol, anhydrous trehalose, isomaltulose, sucrose, galactinol, glucarate-O-phosphoric acid, and turanose. This study reveals that the RLs of mannitol exhibit a significant increase during fermentation, which is in agreement with previous findings that suggest the highest concentration of mannitol is produced by fungi after the fermentation of dark tea, particularly Pu-erh tea, when compared to other teas (Shevchuk et al., 2020).

To verify the metabolism of monosaccharides in fermentation, 13 monosaccharides in tea leaves were measured through HPAEC (Figure 1B). These had levels ranging from 0.01 ± 0.00 mg/g to 60.07 ± 2.14 mg/g, with sum contents of 67.13 ± 2.28 mg/g and 24.83 ± 0.37 mg/g in FT and CK, respectively. The level of fructose was highest in both groups, with contents of 60.07 ± 2.14 mg/g and 19.35 ± 0.21 mg/g in FT and CK, respectively. The contents of eight monosaccharides were increased by fermentation, including trehalose, fucose, rhamnose, arabinose, galactose, glucose, fructose, and raffinose. The contents of sucrose decreased from 3.42 ± 0.10 mg/g to undetectable. The high levels of arabinose, glucose, and sucrose in FT were in accordance with the results of widely targeted metabonomic measurements. Both confirmed the increase in arabinose and glucose as well as the decrease in sucrose.

To summarize, the fermentation of A. luchuensis led to an increase in the levels of soluble sugar, total lignin, total hemicellulose, and monosaccharide, whereas it reduced the amounts of polysaccharide and cell wall cellulose. Previous studies have also reported an increase in the levels of arabinose (Gong et al., 2010), glucose (Wang et al., 2022), mannitol (Zhang et al., 2023), xylitol, sorbitol (Shao et al., 2022), and dulcitol (Li et al., 2022), which is consistent with the findings presented here.

### 3.3 Expression of CAZymes genes in fermentation

The fungal mycelia in fermented tea leaves were collected, and their transcriptomes were sequenced to yield 5.58 × 10^7^ clean reads. A total of 40 genes (FPKM >10) with relatively high expression in fermentation were identified and encoding 16 CAZymes, including β-glucosidase (6 genes), endoglucanase (5 genes), cellobiohydrolase (4 genes), arabinofuranosidase (4 genes), α-amylase (4 genes), pectate lyase (3 genes), α-L-arabinofuranosidase (2 genes), rhamnose carboxylic acid acetylesterase (2 genes), xylanase (2 genes), and β-galactosidase (2 genes).

**Figure 2:**
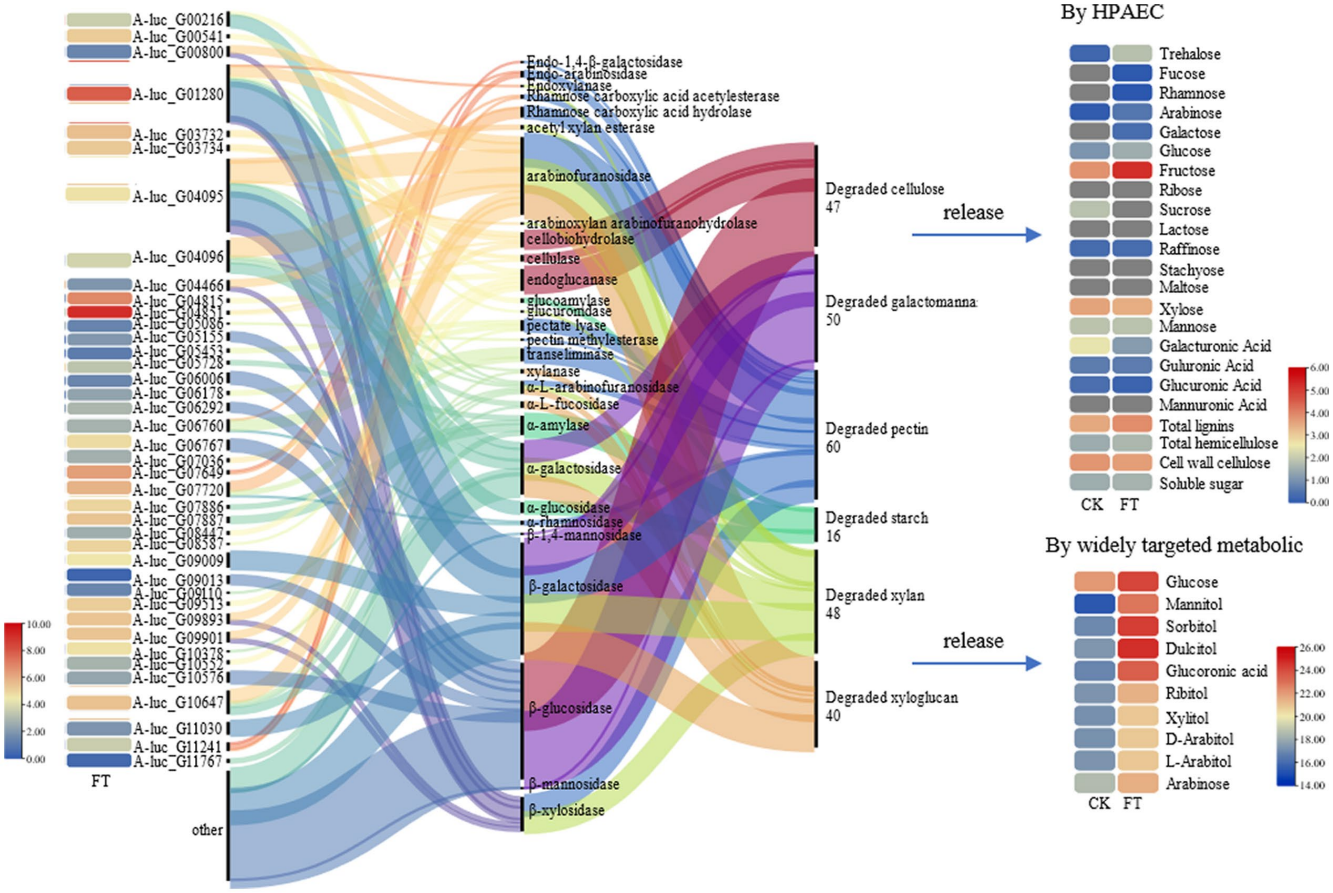
CAZymes gene expression, function, and carbohydrate changes.

Based on the previous analysis of CAZymes in *Aspergillus* (Pel et al., 2007; Coutinho et al., 2009; Culleton et al., 2013, 2014), and the function in UniProt annotation, 16 genes encoding CAZymes were hypothesized to degradation of cellulose, galactomannan, pectin, starch, xylan, and xyloglucan, as well as the release of other substances, such as trehalose, fucose, rhamnose, glucose, mannitol, sorbitol, dulcitol, ribitol, and glucuronic acid (Figure 2). For example, endoglucanase is involved in the degradation of complex natural cellulosic substrates; cellobiohydrolase releases the disaccharide cellobiose from the non-reducing end of the cellulose polymer chain and β-1,4-glucosidases hydrolyzes the cellobiose and other short cello-oligosaccharides into glucose units (Supplementary Table S2).

In summary, *A. luchuensis* P1 is capable of producing CAZymes during fermentation, such as extracellular inulinase, α-amylase, α-glucosidase, arabinofuranosidase, β-galactosidase, pectin methylesterase, and β-mannosidase. These enzymes can break down cellulose, pectin, starch, xylan, and other polysaccharides, resulting in an increase in soluble sugars, D-glucose, D-arabinitol, xylitol, and sucrose. Our findings align with previous studies, which have shown that glucose production increases when bacteria and yeast are combined (Tan et al., 2020). Microbial fermentation also increases the contents of arabinose, galactose, rhamnose, and mannose as reported in a study on the proliferation of microorganisms (Huang et al., 2019). Mannose content increases due to the degradation of microbial exopolysaccharides, as it is a component of such polysaccharides (Tian et al., 2021). The decrease in D-galacturonate content is consistent with a previous report on the lack of microorganisms with transport systems in inoculated fermentation tea (Jeong et al., 2018).

### 3.4 Pathway for carbohydrate metabolism

**Figure 3:**
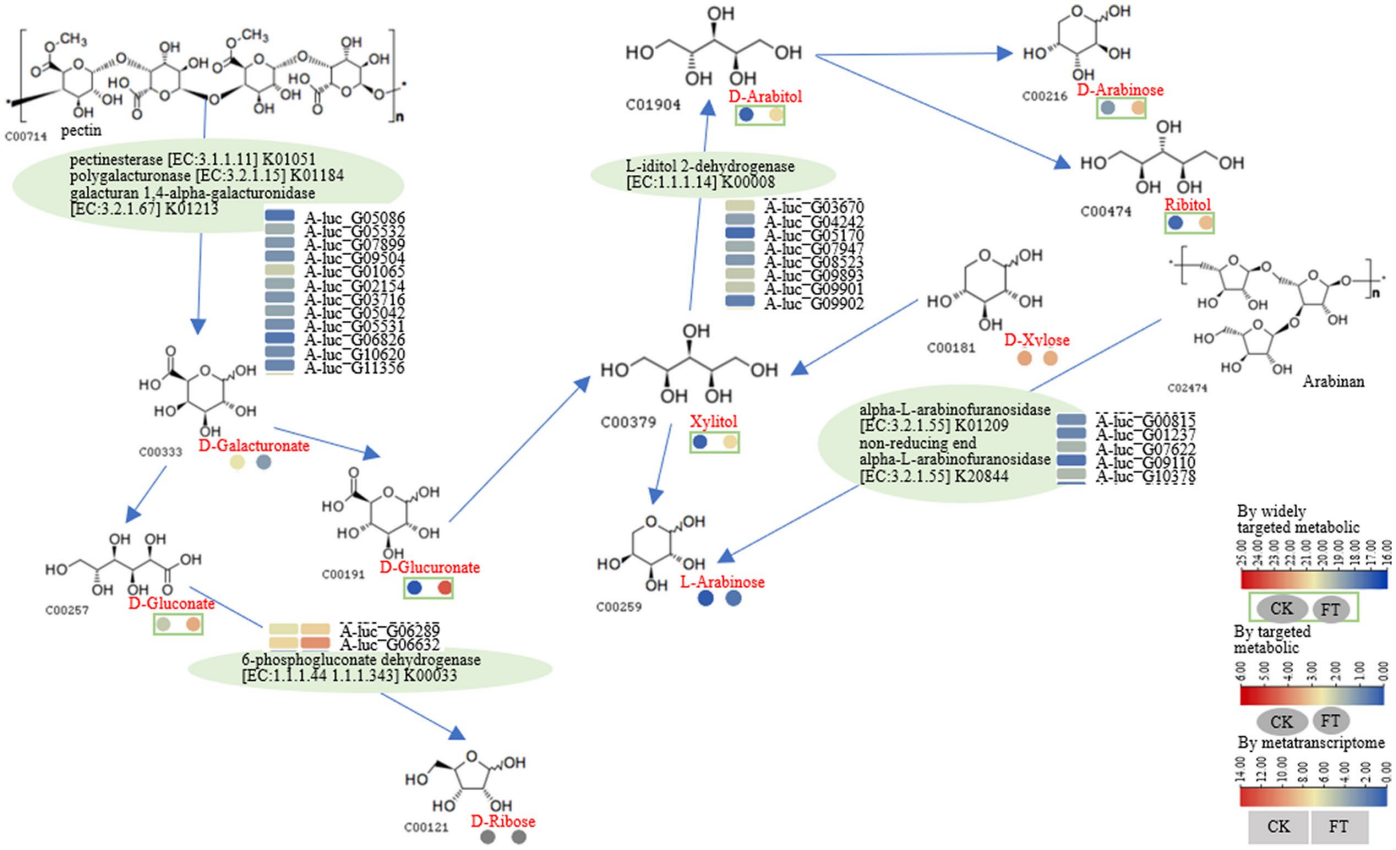
Pathway for the metabolism of D-galacturonate, D-gluconate, xylitol, D-arabitol, and L-arabinose.

After fermentation, the relative content of D-galacturonate decreased nearly 4-fold, whereas the RLs of D-glucuronate, xylitol, D-arabitol, and L-arabinose increased approximately 4- to 32-fold. Ribitol was also increased, whereas D-galacturonate and D-xylose were decreased. L-Iditol 2-dehydrogenase (eight genes), pectinesterase (five genes), polygalacturonase (three genes), galacturan 1,4-α-galacturonidase (four genes), α-L-arabinofuranosidase (three genes), non-reducing-end α-L-arabinofuranosidase (three genes), and 6-phosphogluconate dehydrogenase (two genes) were observed in fermentation. According to the function annotation in the UniProt database, pectinesterase modifies cell walls by demethylesterifying cell wall pectin. Polygalacturonase decomposes pectin and pectic acid, while the arabinose residues in cell wall polysaccharides are primarily degraded by α-L-arabinofuranosidase. The non-reducing-end α-L-arabinofuranosidase hydrolyzes neutral sugars such as arabinogalactan and arabinomannan in the cell wall, promoting the solubilization and degradation of pectin (Supplementary Table S3). A pathway for the metabolism of D-galacturonate, D-gluconate, xylitol, D-arabitol, and L-arabinose is illustrated in Figure 3. It was suggested that pectinesterase (A-luc_G05086, A-luc_G05532, etc.), polygalacturonase (A-luc_G07899, A-luc_G09504, etc.), and galacturan 1,4-α-galacturonidase (A-luc_G01065, A-luc_G02154, etc.) hydrolyzed pectin into D-galacturonate. D-galacturonate was hydrolyzed to D-gluconate by aldonolactonase and further hydrolyzed to D-ribose by 6-phosphogluconate dehydrogenase (A-luc_G06289, A-luc_G06632). L-Iditol 2-dehydrogenase (A-luc_G03670, A-luc_G04242, etc.) dehydrogenated xylitol to D-arabitol, and D-arabitol further released D-arabinose and ribitol. α-L-Arabinofuranosidase (A-luc_G00815, A-luc_G01237, etc.) and non-reducing-end α-L-arabinofuranosidase (A-luc_G10378) hydrolyzed arabinan, releasing L-arabinose (Figure 3).

**Figure 4:**
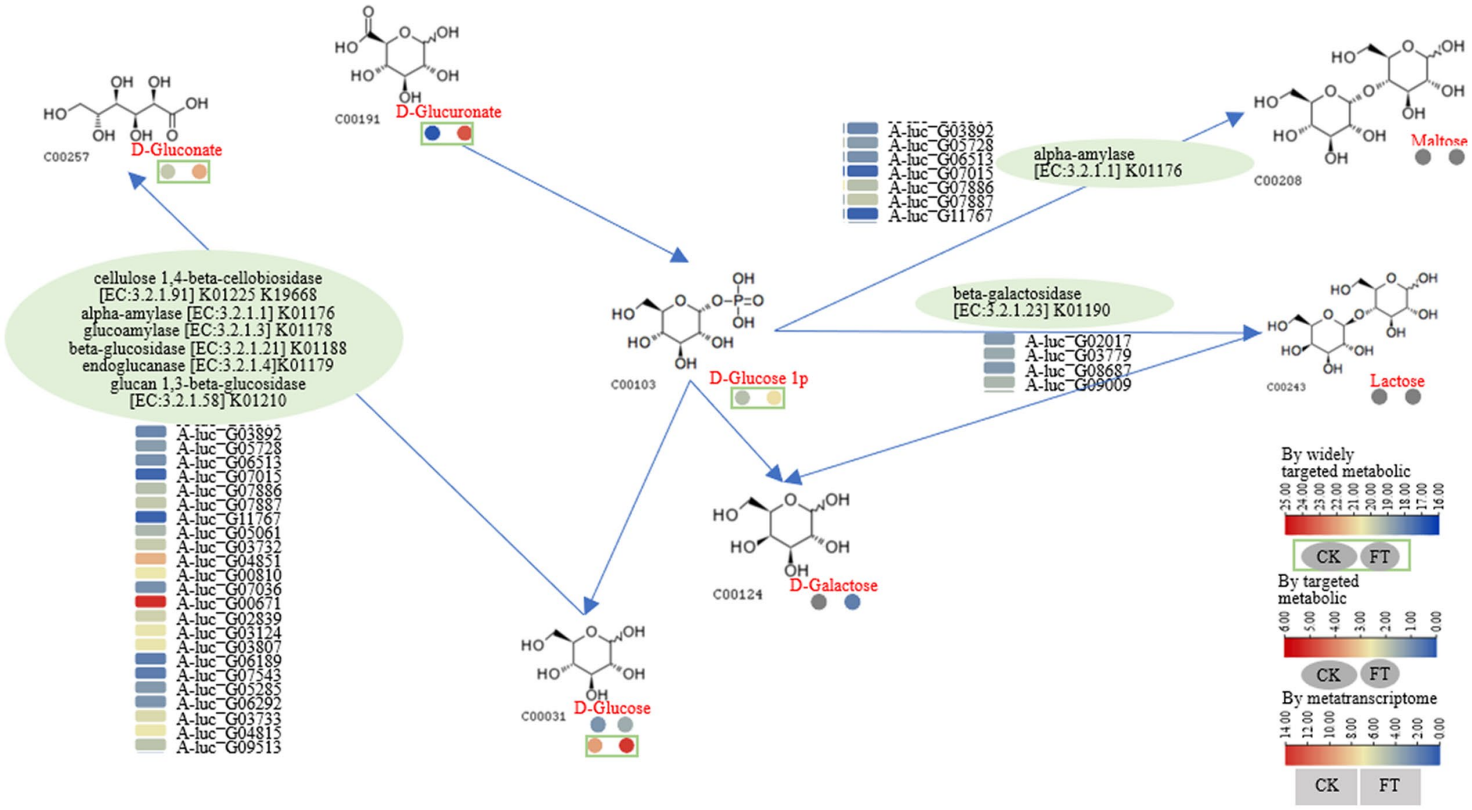
Pathway for metabolism of glucose-1-phosphate, D-gluconate, D-glucose, and D-galactose.

After fermentation, the RLs of glucose-1-phosphate and D-gluconate increased 2.4- and 91-fold, respectively. Furthermore, D-glucose and D-galactose were also increased by 4.1- and 3.7-fold. In the transcriptome, 2 to 12 genes encoding α-amylase, cellulose 1,4-β-cellobiosidase, glucoamylase, endoglucanase, β-glucosidase, β-galactosidase, and glucan-1,3-β-glucosidase were highly expressed (Figure 4). For example, according to the function annotation in UniProt database, these genes may be involved in increasing glucose-1-phosphate and D-gluconate; starch, glycogen, oligosaccharides, or polysaccharide molecules can be hydrolyzed by α-amylase to produce maltose, oligosaccharides, and glucose; Starch and glycogen can also be hydrolyzed by glucoamylase to produce glucose. Therefore, a pathway for the metabolism of glucose-1-phosphate, D-gluconate, D-glucose, and D-galactose was drawn as follows: cellulose 1,4-β-cellobiosidase (A-luc_G00810 and A-luc_G07036), α-amylase (A-luc_G03892, A-luc_G05728, etc.), glucoamylase (A-luc_G05061), β-glucosidase (A-luc_G05285 and A-luc_G06292), endoglucanase (A-luc_G03733, A-luc_G04815, etc.), and glucan-1,3-β-glucosidase (A-luc_G00671, A-luc_G02839, etc.) hydrolyze D-glucose, releasing D-gluconate; α-amylase (A-luc_G03892, A-luc_G05728, etc.) hydrolyzed D-glucose 1p, releasing maltose and β-galactosidase (A-luc_G02017, A-luc_G03779, etc.), and hydrolyzed glucose-1-phosphate, releasing lactose and maltose (Figure 4).

**Figure 5:**
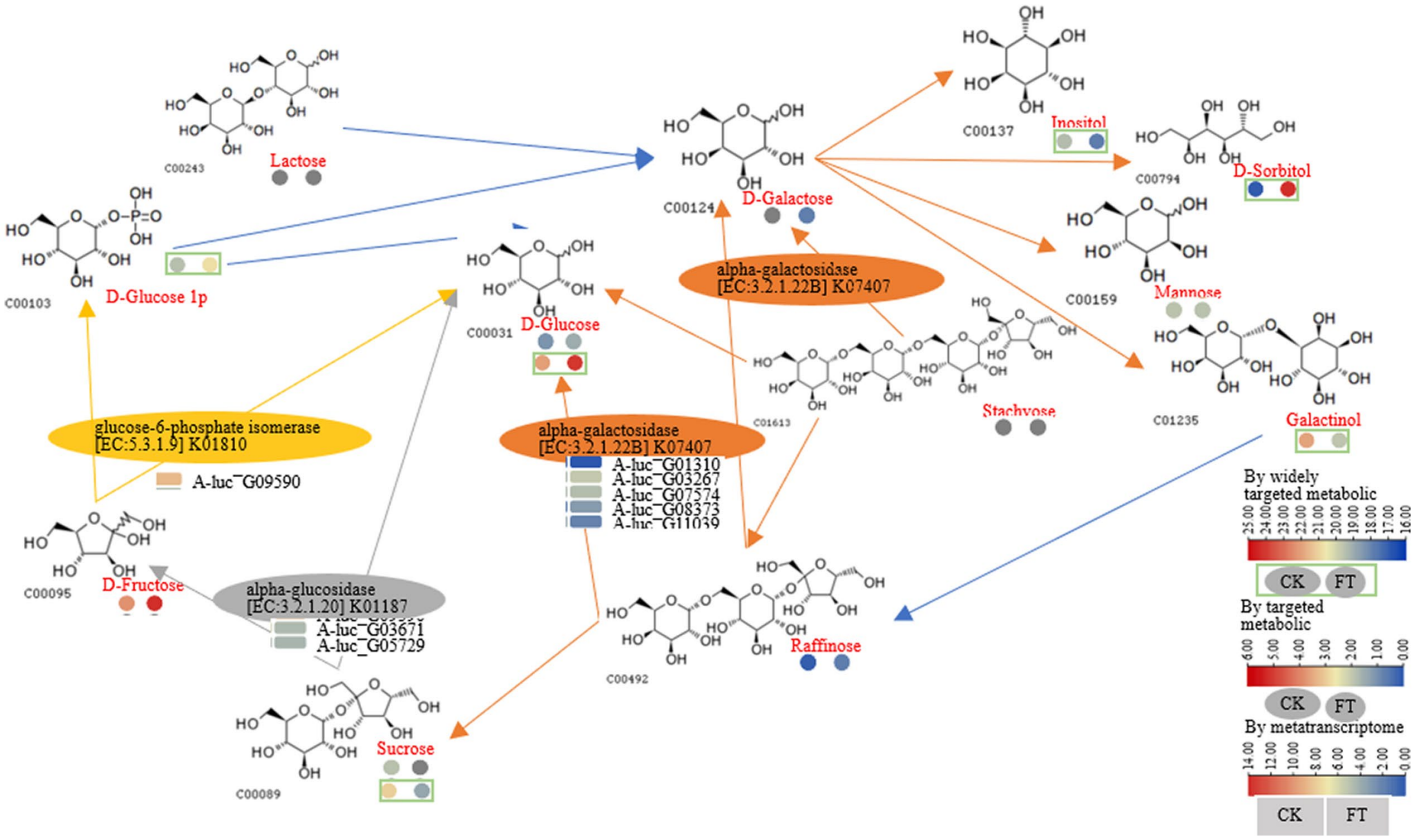
Pathway for starch and sucrose, pentose phosphate, and galactose metabolism.

The widely targeted metabolic analysis identified that the RLs of D-sorbitol and D-glucose increased 145- and 4-fold, respectively. HPAEC revealed that the contents of sucrose (3.4-fold) decreased, whereas the content of trehalose (13.5-fold), fructose (3.1-fold), D-glucose (1.7-fold), and raffinose (1.1-fold) increased. Fucose, rhamnose, and galactose were generated after fermentation. Furthermore, glucose-1-phosphate increased, whereas galactinol and inositol decreased after fermentation. Genes encoding enzymes including α-galactosidase (five genes), α-glucosidase (two genes), and glucose-6-phosphate isomerase (one gene) were suggested to cause these metabolisms of compounds. For example, according to the function annotation in the UniProt database, α-galactosidase can hydrolyze polysaccharides containing α-galactoside bonds; α-Glucosidase can hydrolyze α-glucosides and oligosaccharides (Supplementary Table S3). Together, starch and sucrose metabolism, pentose phosphate pathway, and galactose metabolism were constructed as follows: α-galactosidase (A-luc_G01310, A-luc_G03267, etc.) hydrolyzed D-galactose, releasing inositol; D-sorbitol, mannose, and galactinol. α-Galactosidase hydrolyzed stachyose, releasing D-galactose, D-glucose, and raffinose. α-Galactosidase hydrolyzed raffinose, releasing D-glucose and sucrose; α-Glucosidase (A-luc_G03671 and A-luc_G05729) hydrolyzed sucrose, releasing D-fructose and D-glucose; glucose-6-phosphate isomerase (A-luc_G09590) catalyzed D-fructose, releasing D-glucose and glucose-1-phosphate (Figure 5).

**Figure 6:**
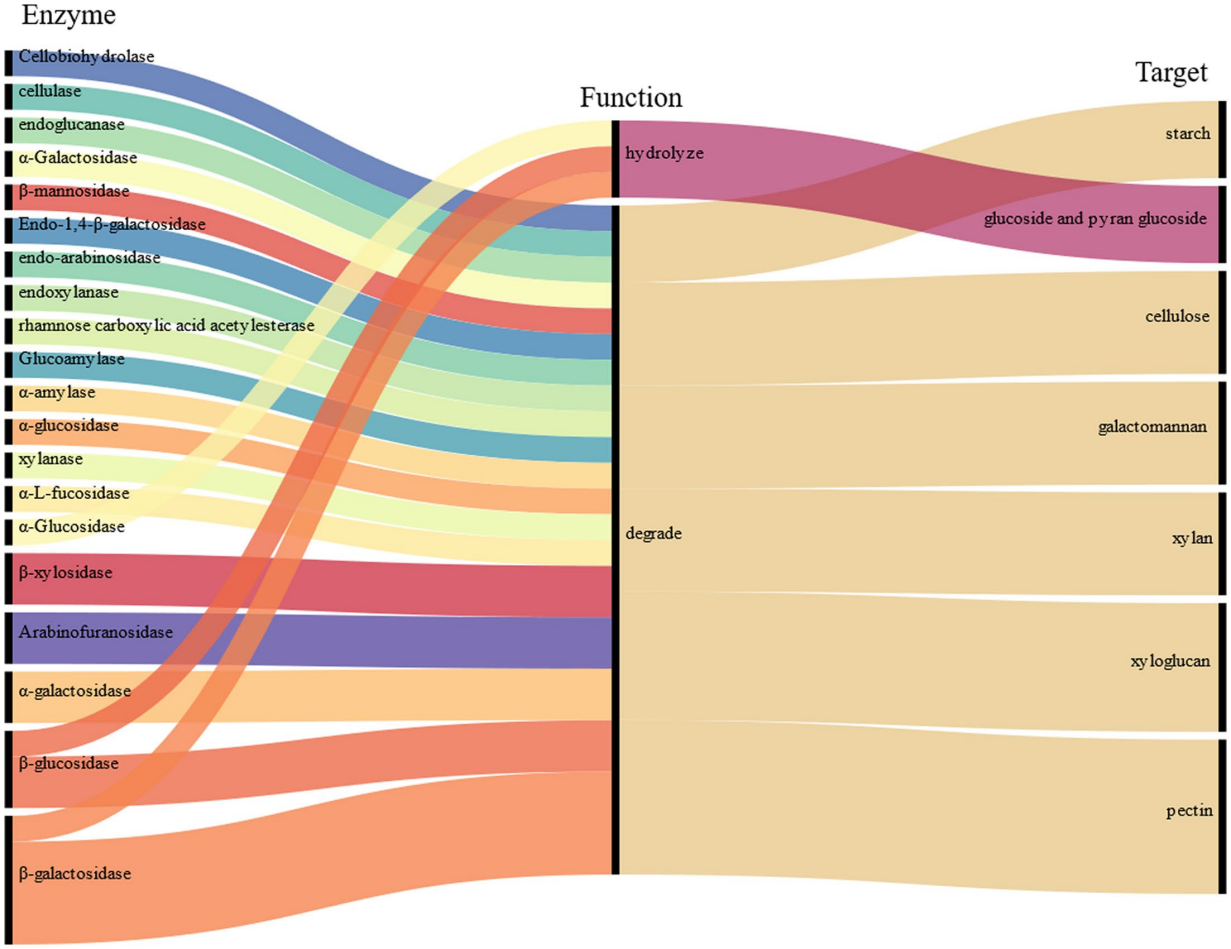
Polysaccharide-related degrading enzyme or hydrolase.

During pile fermentation, sun-dried tea leaves undergo a series of chemical reactions (oxidation, degradation, and condensation), and the composition and content of the main quality-related metabolites such as tea polyphenols, catechins, amino acids, flavonoids and their glycosides, phenolic acids, and alkaloids significantly change (Zhu et al., 2020). The genera *Aspergillus*, *Penicillium*, *Rhizopus*, and *Saccharomyces* are the most frequently identified microorganisms during the process of pile fermentation and in commercial products (He et al., 2023). *A. luchuensis* was isolated from pu-erh tea fermentation and identified as a dominant fungus. In a previous study, gene-encoding enzymes that catalyze the metabolism of phenolic compounds were revealed (Ma et al., 2022). In this study, we found that fermentation by *A. luchuensis* primarily leads to the degradation of polysaccharides and cell wall cellulose, as well as an increase in the content of monosaccharides, soluble sugars, and total lignin; therefore, making a significant contribution to the flavor of RPT; we also observed genes encoding CAZymes highly expressed in fermentation (FPKM >10), which assumed to cause the metabolism of carbohydrates (Figure 6).

## 4 Discussion

The metabolism of carbohydrates by fungi is important in the biological world. Fungi use carbohydrates as a source of energy and carbon, which help them survive and grow. Fungi also play a role in important biogeochemical cycles such as the carbon, nitrogen, and sulfur cycles (Yousaf et al., 2021). These metabolites have applications in areas such as food fermentation, drug development, and industrial production. The study found that *A. luchuensis* produces CAZymes to degrade polysaccharides and cellulose, which has potential applications in fields such as bioengineering or bio-energy and is worth further research.

Pu-erh tea has a unique flavor that is mainly attributed to diverse microorganisms. The traditional fermentation process of Pu-erh tea involves spontaneous microbial solid-state pile fermentation for several weeks under high temperatures and humidity. During this process, the genera *Aspergillus*, *Penicillium*, *Rhizopus*, and *Saccharomyces* are usually identified and isolated microorganisms. These microorganisms are also present in commercial products (He et al., 2023). Pile fermentation changes tea leaves, creating a unique flavor of RPT. Most saccharides and purine alkaloids showed increasing tendency or remained stable (Ma et al., 2021); the change in saccharides was consistent with the research in this study. *Aspergillus* produces abundant hydrolases, such as cellulase, hemicellulase, xylanase, pectinase, and protease. These enzymes hydrolyze cellulose, pectin, and protein substances to form soluble sugars, amino acids, soluble pectin, and other components (Hu et al., 2021). After the inoculation of *A. luchuensis*, the soluble sugar content increased, which has been consistent with previous studies. Tea polyphenols in tea do not have an inhibitory effect on mold. Additionally, some metabolites produced by mold can produce a series of reactions in tea, including degradation, oxidation, methylation, which can improve the quality of tea (Hu et al., 2022). A recent study showed that *A. luchuensis* fermentation increases monosaccharides, soluble sugars, and total lignin. Enzymatic reactions increase specific compounds. These findings provide insight into Pu-erh tea fermentation and flavoring compounds (Wu et al., 2022). A series of reactions such as degradation, glycosylation, deglycosylation, methylation, and oxidative polymerization occurred during solid-state fermentation (Xiao et al., 2022). In a recent study, genes encoding enzymes such as glycoside hydrolases, phenolic acid esterases, laccases, tyrosinases, dehydrogenases, peroxidases, dioxygenases, monooxygenases, decarboxylases, and O-methyltransferases were identified. These enzymes catalyze hydrolysis, oxidation, ring cleavage, hydroxylation, decarboxylation, and O-methylation of phenolic compounds, significantly (*p* < 0.05) changing the phenolic compound composition. Phenolic compounds were found to be degraded through the degradation of aromatic compounds pathways and xenobiotics biodegradation and metabolism pathways (Ma et al., 2022).

This study found that *A. luchuensis* can effectively break down cellulose and increase the number of monosaccharides during tea fermentation. However, there is currently a lack of research on carbohydrate metabolism and mechanisms involved in the processing of Pu-erh tea. This article provides a foundation for further study in this area.

The focus of this study is on the metabolism and mechanism of carbohydrates during the fermentation process of Pu-erh tea. Previous research has shown that carbohydrates are related to the abundance of responsible proteins in varying degrees and potentially contribute to the comprehensive flavor of tea (Wu et al., 2020). Another study found that stress-induced carbohydrates change the flavor formation of oolong tea during the enzymatic-catalyzed process (Wu et al., 2022). Additionally, there are differences in the isolation, identification, and community diversity of microorganisms between tank and pile fermentation (Long et al., 2023). Our study examined the effects of *A. luchuensis* fermentation on tea leaves, including the cell wall, cellulose, pectin, and composition of polysaccharides. We identified the enzymes involved in tea fermentation and discovered the types and amounts of monosaccharides produced from the degradation of cellulose and pectin. Our research provides a foundation for developing processing technologies and utilizing microbial strains to produce dark teas.

## 5 Conclusion

The results of the study indicate that tea fermentation led to an increase in a variety of monosaccharides, including trehalose, fucose, rhamnose, arabinose, galactose, glucose, fructose, and raffinose, as well as other substances such as mannitol, sorbitol, dulcitol, soluble sugar, total hemicellulose, and total lignin in FT (*p* < 0.05). Conversely, the relative content of D-sorbitol, D-glucose, and cell wall cellulose in CK was increased (*p* < 0.05). The study also analyzed the changes in tea carbohydrates and the expression of CAZymes genes of *A. luchuensis*. The results suggest that during fermentation, enzymes such as cellobiohydrolase, cellulase, endoglucanase, β-glucosidase, α-galactosidase, β-galactosidase, and others can break down cellulose, galactomannan, pectin, starch, xylan, and xyloglucan. Additionally, α-glucosidase, β-galactosidase, and β-glucosidase can hydrolyze glucoside and pyran glucoside. These findings provide insight into the metabolism of tea carbohydrates fermented with a dominant fungus and lay the groundwork for further understanding carbohydrate changes in RPT fermentation.

## Data availability statement

The datasets presented in this study can be found in online repositories. The names of the repository/repositories and accession number(s) can be found in the article/Supplementary material.

## Author contributions

RL: Conceptualization, Methodology, Writing – original draft. TW: Methodology, Writing – original draft. NB: Validation, Writing – original draft. QW: Writing – original draft. QC: Writing – original draft. LZ: Supervision, Validation, Writing – original draft. YG: Validation, Visualization, Writing – original draft. BJ: Writing – original draft. YM: Writing – review & editing. MZ: Supervision, Validation, Writing – review & editing.

## Footnotes

^1^ https://www.metaboanalyst.ca/
